# Optogenetic induced epileptiform activity in a model human cortex

**DOI:** 10.1186/s40064-015-0836-7

**Published:** 2015-04-01

**Authors:** Prashanth Selvaraj, Jamie W Sleigh, Heidi E Kirsch, Andrew J Szeri

**Affiliations:** Department of Mechanical Engineering, University of California, Berkeley, 94720 CA USA; Waikato Clinical School, University of Auckland, Hamilton, New Zealand; Departments of Neurology and Radiology and Biomedical Imaging, University of California, San Francisco, 94143 CA USA; Center for Neural Engineering and Prosthesis, University of California, Berkeley, 94720 CA USA

**Keywords:** Meso-scale cortical model, Epiletic seizure propagation, Optogenetic stimulation

## Abstract

**Background:**

Cortical stimulation plays an important role in the study of epileptic seizures. We present a numerical simulation of stimulation using optogenetic channels expressed by excitatory cells in a mean field model of the human cortex.

**Findings:**

Depolarising excitatory cells in a patch of model cortex using Channelrhodpsin-2 (ChR2) ion channels, we are able to hyper-excite a normally functioning cortex and mimic seizure activity. The temporal characteristics of optogenetic channels, and the ability to control the frequency of synchronous activity using these properties are also demonstrated.

**Conclusions:**

Optogenetics is a powerful stimulation technique with high spatial, temporal and cell-type specificity, and would be invaluable in studying seizures and other brain disorders and functions.

## Introduction

In seizure research, cortical stimulation has been used as a means to study the pre-seizure state, epileptogenesis, the epileptic state and seizure control. In vivo stimulation using radial electric fields (Richardson et al. 2003), and high frequency (Su et al. 2008) and low frequency (Jerger and Schiff 1995) *in vitro* electrical stimulation are some of the methods that have been used to modulate epileptiform activity. However, while electrical stimulation has excellent temporal resolution, it does not offer a means to target specific cell populations in brain regions at multiple different spatial scales. Optogenetics, on the other hand, offers excellent temporal and spatial resolution. Targeted optogenetic stimulation of neurons (Cardin et al. 2010) and the effect of optogenetics on neural circuitry (Zhang et al. 2007) point to the novel use of this method as a highly specific cell stimulation technique. Furthermore, optogenetics has been used to inhibit epileptic seizures in *in vivo* and *in vitro* experiments in animals (Krook-Magnuson et al. 2013; Paz et al. 2013; Tønnesen et al. 2009), and the specificity of targeting this technology offers would be invaluable in studying the role of individual neurons and neural circuits in epilepsy. Optogenetic technology involves genetically modifying specific neurons to express light sensitive ion channels without changing the cell’s underlying physiology, and requires further rigorous testing before it is deemed safe for use in humans. However, the efficacy of this stimulation technique in inhibiting seizures has been examined in a mathematical model of human cortex (Selvaraj et al. 2014). In this article, we present a study of the potential for optogenetics as a method to induce seizures in a model human cortex by depolarising the excitatory population in a patch of the model cortex using ChR2 channels.

First, we demonstrate the propagation of seizure waves through a normally functioning cortex that is stimulated using optogenetic channels. Next, we look at the effect of illumination intensity on the onset and frequency of the induced seizure waves. Finally, the effect of using pulsed illumination and its role in varying the frequency of synchronous activity is discussed.

## Seizure initiation

We use the meso-scale cortical model developed by Liley et al. (2001) to simulate the dynamics of a human cortex. The non-dimensionalised form of this model can be found in the appendix.

To study the effects of optogenetic stimulation on the cortex, we use the four state model of Channelrhodopsin-2 (ChR2) proposed in (Grossman et al. 2011). A meso-scale version of this model described in the appendix, was combined with the above mentioned cortical model by modifying the inhibitory cell population to express light sensitive ChR2 ion channels (Selvaraj et al. 2014). Here, the excitatory population of the cortical model expresses ChR2 channels, which changes the equation describing the dynamics of the mean soma potential of the excitatory population to:

(1) $$  \frac{\partial \tilde{h}_{e}}{\partial \tilde{t}} = 1 - \tilde{h}_{e} + \Gamma_{e}\left({h_{e}^{0}} - \tilde{h}_{e}\right)\tilde{I}_{ee} + \Gamma_{i}\left({h_{i}^{0}} - \tilde{h}_{e}\right)\tilde{I}_{ie} - u.  $$

The term *u* is the stimulation applied to the excitatory population, and is given by,

(2) $$  u = \tilde{h}_{e}.G_{ChR2}.R_{m}.  $$

where $\tilde {h}_{e}$ is the membrane potential for the excitatory population, *R*_*m*_ is the membrane resistance of the cells, and the conductance of ChR2 channels, *G*_*ChR*2_, is defined as

(3) $$  G_{ChR2} = G_{max}. g_{ChR2}. \frac{\left(1 - exp(-h_{e}/U_{0})\right)}{h_{e}/U_{1}}. N_{ChR2},  $$

where *g*_*ChR*2_ is the total conductance of the optogenetic channels in the *O*1 and *O*2 states. *U*_0_ and *U*_1_ are empirical constants and *N*_*ChR*2_ is the number of ChR2 channels per representative neuron. The expression for *g*_*ChR*2_, and values and explanations for all parameters can be found in (Selvaraj et al. 2014).

## Results and discussion

**Figure 1:**
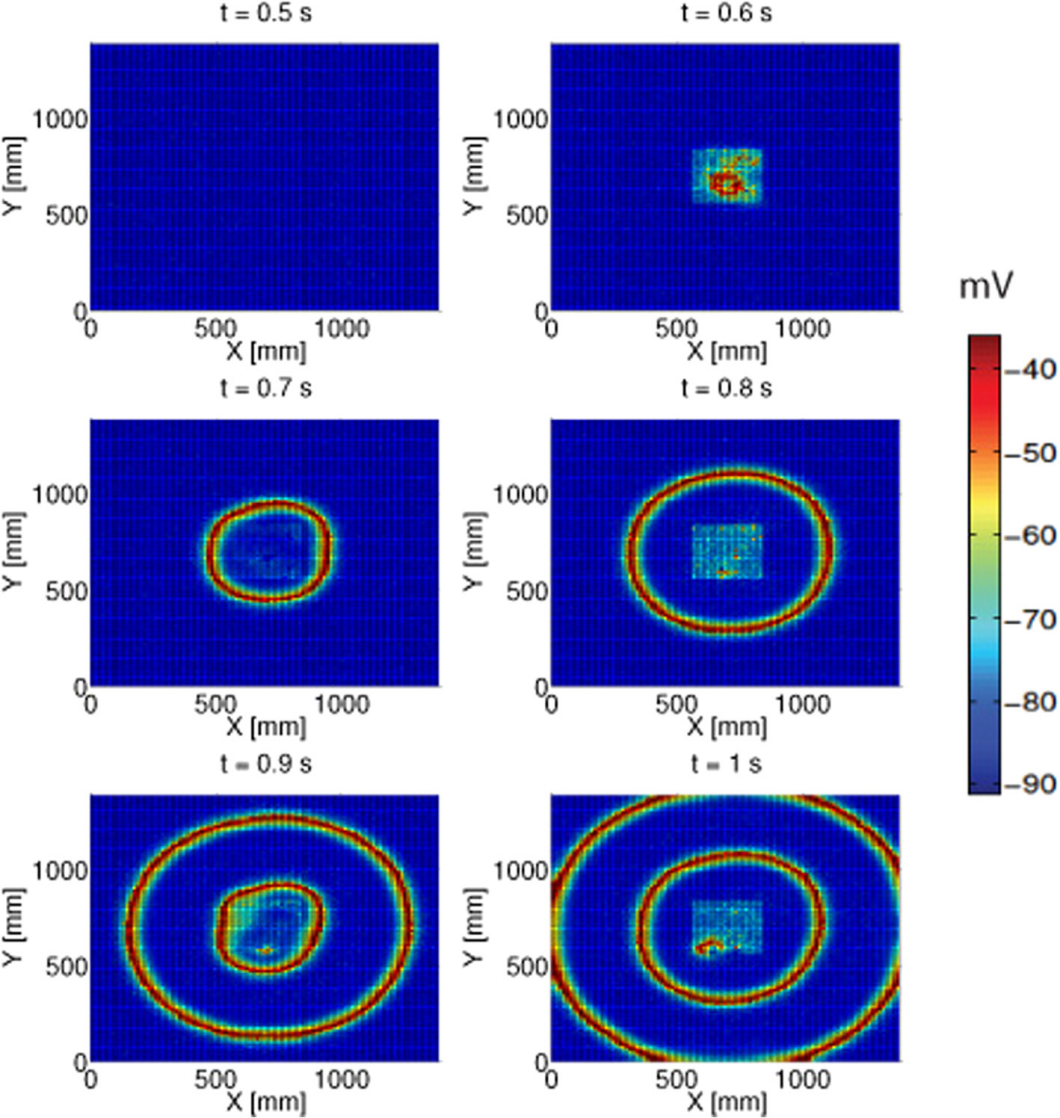
Propagation of seizure waves in a 2D model of a normally functioning human cortex when optogenetic stimulation is applied to a 280×280 mm^2^ patch in the centre of the cortex. Snapshots are taken from time t=0.5 s to t=1 s. Normal cortical function is characterized here by the baseline parameters of (Kramer et al. 2006) with P_*ee*_=11.0 and *Γ*_*e*_=0.0012. The cortex is stimulated with a constant light intensity of 50 mW /mm^2^ at 0.5 s.

We now present results obtained by optogenetic stimulation of a portion of model cortex of dimensions 1400×1400 mm^2^, which is divided into 100×100 cells or representative neurons^a^. We stimulate an area of model cortex with spatial scale of the order of clinical recordings. In Figure 1, a square region in the middle of the cortex of approximate dimensions 280×280 mm^2^ (1/25 the total area of the cortex) is modified to express ChR2 ion channels in the excitatory population, and this region is illuminated with light of a constant 50 mW /mm^2^ intensity starting at 0.5 s. An ion channel density of 10^9^ChR2^′^s/*m*^2^ is used to keep illumination intensities within physiologically permissible values and still ensure optimal stimulation of the cortex.

Figure 1 depicts the birth of a seizure-like wave within the square patch of stimulated cortex. Neighbouring regions are then excited by the outward propagation of travelling seizure waves, and the effects of the stimulation spread to the entire cortical area. Hyper-excitation in (Selvaraj et al. 2014) was achieved by increasing subcortical inputs to the excitatory population throughout the model cortex. This meant the entire cortex was on the verge of seizing, and a small increase in subcortical inputs to a column could cause a region of cortex to seize. These seizures originated from a cortical column, and propagated outwards in spiral waves.

Here, spatially uniform travelling waves are formed by the equal hyper-excitation of cortical macrocolumns within a patch of cortex using optogenetic channels, and subcortical inputs do not play as important a role in starting seizures as illumination intensity increases.

It should be noted here that the entire model cortex is functioning normally, with inhibitory and excitatory inputs of comparable magnitudes balancing each other out. However, when adequate optogenetic stimulation is applied to the excitatory population, it depolarises these cells, increasing their mean soma potential, which immediately increases their firing rate. The local excitatory and long range contributions to post synaptic activation are increased because of the higher firing rate, producing a further rise in the mean soma potential of both populations, which ultimately leads to even higher firing rates in both excitatory and inhibitory cells. Increases in inhibitory firing rates trail excitatory ones within a macrocolumn by 1-2 ms for two reasons. One, stimulation is applied directly to the excitatory population, and two, there are time delays associated with synaptic transmission. Spatial connectivity between columns transfers synchronous activity to neighbouring columns of neurons outside the stimulated cortical patch, exciting them into synchronous states facilitating propagation of seizure activity. As stated earlier, only neurons within the stimulated area are hyperexcited while the rest of the cortex is functioning normally. This constrains the minimum stimulated area necessary to excite the rest of the cortex into an epileptic state to ∼4% of the total area of the cortex. Smaller stimulated regions are not able to support seizure activity for cortical and optogenetic parameters mentioned above.

**Figure 2:**
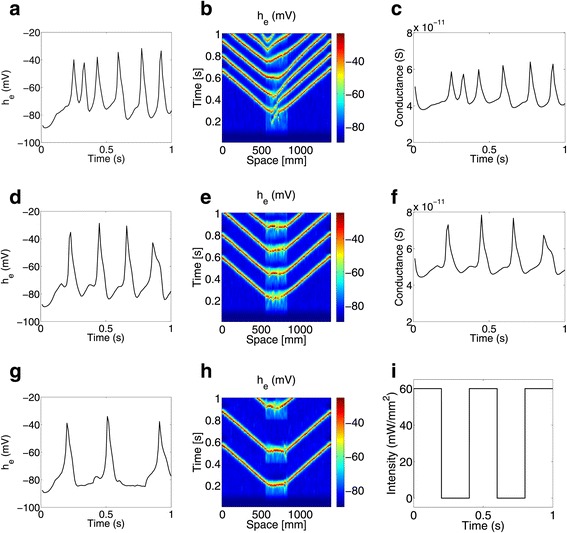
Optogenetic stimulation of a normally functioning cortex from *t*=0 s.**(a-c)** represent the variation of *h*_*e*_ with time, travelling waves in a 1-D slice of the 2-D domain, and the variation of *G*_*ChR*2_ with time, respectively, using a constant intensity of 30 mW /mm^2^. **(d-f)** show the same features using a constant intensity of 60 mW /mm^2^. **(g-i)** show the variation of *h*_*e*_ at a point, a 1-D slice of the 2-D domain and the pulsed illumination profile with a maximum intensity of 60 mW /mm^2^.

In Figure 2, we look at the variation of mean soma potential of the excitatory population at a point within the stimulated patch of cortex, and take a one dimensional cross section of the two dimensional spatial domain to study how different illumination intensities and illumination profiles lead to travelling seizure waves of varying frequencies. For all three illumination profiles presented in Figure 2, stimulation is turned on at t = 0 s. Figures 2a and 2d show the variation of mean soma potential with time when the cortical patch is constantly illuminated with intensities of 30 mW /mm^2^ and 60 mW /mm^2^, respectively, while Figure 2g uses a 200 ms on 200 ms off pulsed illumination of 60 mW /mm^2^ intensity as shown in Figure 2i. Figures 2b, 2e and 2h depict travelling waves in a one dimensional slice of the two dimensional domain, and comprises both stimulated cortex between 560 mm and 840 mm, and normally functioning cortex.

Higher illumination intensities result in higher conductances, as shown in Figures 2c and 2f, resulting in cells being depolarised more quickly. For a given illumination intensity, pulsed light can reduce the frequency of seizure waves because the stimulatory input rapidly drops to zero when light is turned off, sending the cortex back to a normally functioning state. In other words, increasing the time of no illumination when using a pulsed light source decreases seizure frequency. This difference can be seen between Figure 2d, where constant illumination is used, and Figure 2g, where pulsed illumination is used. Counter-intuitively, though, while a higher intensity depolarises cells more quickly, it reduces the frequency of seizure waves as seen by comparing Figures 2a and 2d. One possible explanation is that the rate of change of mean soma potential given in equation 1 is lower for higher intensities because the stimulation term, u, is always positive on account of using ChR2, a cation pump. A higher rate of change decreases the time required to change from a lower to a higher firing rate and back. In other words, the rate of change of firing rate increases, which results in higher frequency oscillations.

We turn to bifurcation analysis to give us a picture of what happens in the cortex when illumination of a certain intensity is used. To perform a bifurcation analysis, we turn off the stochastic and spatial terms in the cortical model to obtain an underlying set of deterministic ordinary differential equations (ODEs), which helps us gain insight into the complete system of stochastic partial differential equations (SPDEs) that describe the mesoscale cortical model intuitively. It has been shown that Hopf bifurcations in the dimensionless ODEs can correspond to travelling waves in the SPDE system (Kramer et al. 2007). Here, we combine this ODE system with the optogenetic model to study the dynamics of the combined system.

**Figure 3:**
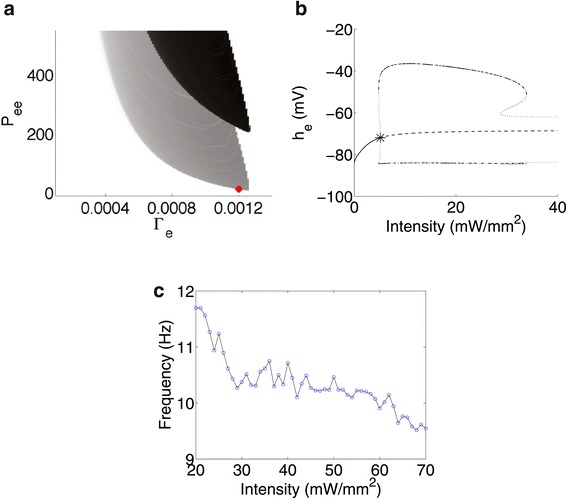
Seizures with the ODE model and frequency response of the SPDE system.**(a)** effect of optogenetic stimulation on oscillatory behaviour in the *Γ*_*e*_ - *P*_*ee*_ parameter space, using a 60 mW /mm^2^ illumination intensity (grey) and in an unstimulated cortex (black). The red dot indicates *Γ*_*e*_ = 0.0012 and *P*_*ee*_ = 11.0, which are used in the full SPDE system. **(b)** Bifurcation diagram for the underlying deterministic ODE system showing the variation of *h*_*e*_ for different illumination intensities. Dashed and solid lines indicate unstable and stable fixed points, respectively. Maximum and minimum values of *h*_*e*_ during stable (dot-dashed) and unstable (dashed) limit cycles arising from a subcritical Hopf bifurcation (asterisk) are also shown. **(c)** Frequency of seizure waves for a given illumination intensity.

Figure 3a shows the values of *Γ*_*e*_ and *P*_*ee*_ that cause oscillatory behaviour in the ODE model when not stimulated (black) and when stimulated (grey) by optogenetic channels using an illumination intensity of 60 mW /mm^2^. The red dot in the figure is located at *Γ*_*e*_ = 0.0012 and *P*_*ee*_ = 11.0, which are used in the full SPDE system simulations shown in Figures 1 and 2. These values lie well outside the region of epilepsy in the unstimulated cortex. The seizure prone area in the parameter space is vastly increased when optogenetic stimulation is applied. It has been observed, but not shown here, that this area is slightly larger with less distinct boundaries for the SPDE system owing to stochasticity.

A bifurcation analysis of the ODE system yields a bifurcation diagram depicting the salient features for the mean soma potential of the excitatory population, *h*_*e*_, for different illumination intensities as shown in Figure 3b. We use *P*_*ee*_ = 11.0 and *Γ*_*e*_ = 0.0012 for this analysis. Solid lines indicate stable fixed points, while dashed lines represent unstable ones. Dot-dashed lines and dotted lines indicate maximum and minimum values of *h*_*e*_ achieved during stable and unstable limit cycles, respectively. The asterisk represents a subcritical Hopf bifurcation at 5.1352 mW /mm^2^, which gives rise to an unstable limit cycle. At 4.7908 mW /mm^2^ the limit cycle stabilises after going through a saddle node bifurcation, and becomes unstable again after going through another saddle node bifurcation at 33.8682 mW /mm^2^. While Figure 3b is truncated at 40 mW /mm^2^, the limit cycle remains unstable and does not terminate well beyond 100 mW /mm^2^, which is around the limit of physiologically acceptable illumination intensities^b^. This suggests the combined cortical-optogenetic model is unable to support stable oscillations past 34 mW /mm^2^, and at higher intensities, we might be observing a succession of independent columnar spiking events and not entrained spiking seen during stable oscillatory behaviour. In the case of an individual spike in mean soma potential, a cortical column relaxes to its resting potential before firing again. This takes longer to produce a spike than the case with continuous oscillatory behaviour, where a column can be excited to spike again before reaching resting state. This explains why lower frequency seizure waves are observed when higher illumination intensities are used, a trend which is shown in Figure 3c after averaging frequency over multiple simulations of the full SPDE system. For lower intensities, subcortical inputs may aid in exciting or suppressing the system, so the probability of producing seizures decreases for intensities lower than 25 mW /mm^2^. For higher intensities, the system will almost always produce seizures, but the frequency of seizure waves is dependent on the magnitude of subcortical and long range inputs. Overall, however, we observe a tendency to produce lower seizure wave frequencies for higher illumination intensities.

## Conclusion

While this study explores the use of cation pumps in the excitatory population of the meso-scale cortical model, an equally interesting alternative using anion pumps to suppress the firing of inhibitory neurons could be investigated. The wide variety of illumination options, light activated ion channels, and their temporal and spatial specificity make a strong case for consideration of optogenetics as a cortical stimulation modality in seizure research.

## Endnotes

^a^ The average human cortex has dimensions of 500×500 mm^2^ if it were laid open like a sheet. However, to remain consistent with previous work, and because dynamics in this model of undifferentiated cortex are scale-free, we have used a larger cortical domain to illustrate seizure waves.

^b^ Prolonged exposure (>0.5 s) at an intensity of 100 mW /mm^2^ caused significant tissue damage in animal models (Cardin et al. 2010).

## Appendix

The non-dimensional meso-scale model was first stated in (Kramer et al. 2006), where values and explanations for all variables and constants of the model can be found. For convenience, we state the equations here.

(A1) $$\begin{array}{*{20}l} &{}\frac{\partial \tilde{h}_{e}}{\partial \tilde{t}} = 1 - \tilde{h}_{e} + \Gamma_{e}\left({h_{e}^{0}} - \tilde{h}_{e}\right)\tilde{I}_{ee} + \Gamma_{i}\left({h_{i}^{0}} - \tilde{h}_{e}\right)\tilde{I}_{ie} \end{array} $$

(A2) $$\begin{array}{*{20}l} &{}\frac{\partial \tilde{h}_{i}}{\partial \tilde{t}} = 1 - \tilde{h}_{i} + \Gamma_{e}\left({h_{e}^{0}} - \tilde{h}_{i}\right)\tilde{I}_{ei} + \Gamma_{i}\left({h_{i}^{0}} - \tilde{h}_{i}\right)\tilde{I}_{ii} \end{array} $$

(A3) $$\begin{array}{*{20}l} &{}\left(\frac{1}{T_{e}}\frac{\partial}{\partial \tilde{t}}+1 \right)^{2}\tilde{I}_{ee} = N^{\beta}_{e}\tilde{S}_{e}\left[\tilde{h}_{e} \right] + \tilde{\phi}_{e} + P_{ee} + \tilde{\Gamma}_{1} \end{array} $$

(A4) $$\begin{array}{*{20}l} &{}\left(\frac{1}{T_{e}}\frac{\partial}{\partial \tilde{t}}+1 \right)^{2}\tilde{I}_{ei} = N^{\beta}_{e}\tilde{S}_{e}\left[\tilde{h}_{e} \right] + \tilde{\phi}_{i} + P_{ei} + \tilde{\Gamma}_{2} \end{array} $$

(A5) $$\begin{array}{*{20}l} &{}\left(\frac{1}{T_{i}}\frac{\partial}{\partial \tilde{t}}+1 \right)^{2}\tilde{I}_{ie} = N^{\beta}_{i}\tilde{S}_{i}\left[\tilde{h}_{i} \right] + P_{ie} + \tilde{\Gamma}_{3} \end{array} $$

(A6) $$\begin{array}{*{20}l} &{}\left(\frac{1}{T_{i}}\frac{\partial}{\partial \tilde{t}}+1 \right)^{2}\tilde{I}_{ii} = N^{\beta}_{i}\tilde{S}_{i}\left[\tilde{h}_{i} \right] + P_{ii} + \tilde{\Gamma}_{4} \end{array} $$

(A7) $$\begin{array}{*{20}l} &{}\left(\frac{1}{\lambda_{e}}\frac{\partial}{\partial \tilde{t}}+1 \right)^{2}\tilde{\phi}_{e} = \frac{1}{{\lambda_{e}^{2}}}\nabla^{2}\tilde{\phi}_{e} + \left(\frac{1}{\lambda_{e}}\frac{\partial}{\partial \tilde{t}}+1 \right) N^{\alpha}_{e}\tilde{S}_{e}\left[\tilde{h}_{e} \right] \end{array} $$

(A8) $$\begin{array}{*{20}l} &{}\left(\frac{1}{\lambda_{i}}\frac{\partial}{\partial \tilde{t}}+1 \right)^{2}\tilde{\phi}_{i} = \frac{1}{{\lambda_{i}^{2}}}\nabla^{2}\tilde{\phi}_{i} + \left(\frac{1}{\lambda_{i}}\frac{\partial}{\partial \tilde{t}}+1 \right) N^{\alpha}_{i}\tilde{S}_{e}\left[\tilde{h}_{e} \right] \end{array} $$

All variables have been non dimensionalised and are functions of time $\tilde {t}$, and the two spatial dimensions $\tilde {x}$ and $\tilde {y}$. The subscripts *e* and *i* represent excitatory and inhibitory populations respectively, and variables with two subscripts represent the transfer of energy from one population to another. The mean soma potential for a neuronal population is represented by the $\tilde {h}$ state variable, $\tilde {I}$ represents the postsynaptic activation due to local, long-range, and subcortical inputs. $\tilde {\phi }$ represents long range (corticocortical) inputs.

The equations describing the dynamics of the optogenetics are,

(A9) $$\begin{array}{*{20}l} &\frac{{dN}_{O1}}{dt} = K_{a1}.N_{C1} - (K_{d1}+e_{12}).N_{O1} + e_{21}.N_{O2}  \end{array} $$

(A10) $$\begin{array}{*{20}l} &\frac{{dN}_{O2}}{dt} = K_{a2}.N_{C2} + e_{12}.N_{O1} + (K_{d2}+e_{21}).N_{O2}  \end{array} $$

(A11) $$\begin{array}{*{20}l} &\frac{{dN}_{C2}}{dt} = K_{d2}.N_{O2} - (K_{a2}+K_{r}).N_{C2}, \end{array} $$

where *N*_*Oi*_ and *N*_*Ci*_ represent the fraction of channels in the open and closed states, respectively. *K*_*ai*_ are the rates of transition from the closed states, *C*1 and *C*2, to the open states *O*1 and *O*2 respectively. Conversely, *K*_*di*_ are the closing rates from the open states to the closed states. *K*_*r*_ is the thermal recovery rate from *C*2 to *C*1. *e*_12_ and *e*_21_ are the transition rates from *O*1 to *O*2 and vice versa. The values for all rate constants can be found in table (Selvaraj et al. 2014).
